# The Epidemic Characteristics and Changing Trend of Hemorrhagic Fever with Renal Syndrome in Hubei Province, China

**DOI:** 10.1371/journal.pone.0092700

**Published:** 2014-03-21

**Authors:** Yi-Hui Zhang, Liang Ge, Li Liu, Xi-Xiang Huo, Hai-Rong Xiong, Yuan-Yuan Liu, Dong-Ying Liu, Fan Luo, Jin-Lin Li, Jia-Xin Ling, Wen Chen, Jing Liu, Wei Hou, Yun Zhang, Hong Fan, Zhan-Qiu Yang

**Affiliations:** 1 State Key Laboratory of Virology, Institute of Medical Virology, School of Medicine, Wuhan University, Wuhan, Hubei, PR China; 2 Tianjin Institute of Surveying and Mapping, Tianjin, PR China; 3 Hubei Provincial Center for Disease Control and Prevention, Wuhan, Hubei, PR China; 4 Institute of Military Medical Sciences, Nanjing Command, Nanjing, PR China; 5 State Key Laboratory of Information Engineering in Survey, Mapping and Remote Sensing, Wuhan University, Wuhan, Hubei, PR China; National Institutes of Health. National Institute of Allergy and Infectious Diseases, Division of Clinical Research, United States of America

## Abstract

**Background:**

Hemorrhagic fever with renal syndrome (HFRS) is caused by different hantaviruses within the *Bunyaviridae* family. HFRS is a fulminant, infectious disease that occurs worldwide and is endemic in all 31 provinces of China. Since the first HFRS case in Hubei Province was reported in 1957, the disease has spread across the province and Hubei has become one of the seriously affected areas in China with the greatest number of reported HFRS cases in the 1980's. However, the epidemic characteristics of HFRS in Hubei are still not entirely clear and long-term, systematic investigations of this epidemic area have been very limited.

**Methods:**

The spatiotemporal distribution of HFRS was investigated using data spanning the years 1980 to 2009. The annual HFRS incidence, fatality rate and seasonal incidence between 1980 and 2009 were calculated and plotted. GIS-based spatial analyses were conducted to detect the spatial distribution and seasonal pattern of HFRS. A spatial statistical analysis, using Kulldorff's spatial scan statistic, was performed to identify clustering of HFRS.

**Results:**

A total of 104,467 HFRS cases were reported in Hubei Province between 1980 and 2009. Incidence of and mortality due to HFRS declined after the outbreak in 1980s and HFRS cases have been sporadic in recent years. The locations and scale of disease clusters have changed during the three decades. The seasonal epidemic pattern of HFRS was characterized by the shift from the unimodal type (autumn/winter peak) to the bimodal type.

**Conclusions:**

Socioeconomic development has great influence on the transmission of hantaviruses to humans and new epidemic characteristics have emerged in Hubei Province. It is necessary to reinforce preventative measures against HFRS according to the newly-presented seasonal variation and to intensify these efforts especially in the urban areas of Hubei Province.

## Introduction

Hemorrhagic fever with renal syndrome (HFRS) is a zoonosis that is caused by different hantaviruses from the family *Bunyaviridae* [1], [2], [3]. HFRS was first recognized in China in the northeast in 1931 and has been prevalent in many other parts of China since 1955 [4]. At present, HFRS is endemic in all 31 provinces of the People's Republic of China, where it is a significant public health problem with 20,000–50,000 human cases diagnosed annually [5], [6]. In China, HFRS is mainly caused by two types of hantaviruses, the Hantaan virus (HTNV) and the Seoul virus (SEOV), each of which co-evolved within a distinct rodent host. HTNV is carried by striped field mice (*Apodemus agrarius*), whereas SEOV is associated with Brown Norway rats (*Rattus norvegicus*) and causes a less severe form of HFRS [7], [8], [9]. The incidence of HFRS is linked to the hantavirus species and is influenced by spatial distribution and infection in animal hosts [9], [10]. It is also thought that human activities and natural factors are related to the occurrence and epidemic of hantaviruses [11], [12], [13].

The incidence of HFRS is highly variable at both the provincial and county level. Although the incidence of and mortality due to HFRS have declined in China during the past decade, the disease has been detected in areas where no cases had been reported before [14], [15]. The endemic areas have extended from rural to urban areas and even into city centers [4], [16]. HFRS incidence has been increasing in some larger cities, such as Beijing and Shenyang [4], [17]. Zuo *et al.* identified three HFRS ‘hotspots’ in Beijing and suggested that the high risk was associated with genetic variation and recombination in SEOV strains. A lot of research has been focused on genetic variability and novel variants of hantaviruses. The Amur virus (AMRV) and Puumala virus (PUUV) have recently been detected in northeastern China [18], [19]. It is evident that new epidemiological features of hantavirus infections have emerged in China, however, most epidemiological studies have focused on regions that are newly or severely affected; long-term systematic investigations focusing on one epidemic area have been very limited.

Hubei Province, located along the Yangtze River in the central-south China, is an area that has been severely affected by HFRS for a number of years. HFRS cases in Hubei Province were first reported in a suburb of Wuhan County in1957. Since then, the disease has spread across the province. In fact, Hubei Province was the area of the country that was most severely affected by HFRS in the early 1980s [20], [21]. A large number of investigations have been done on the serological and molecular analysis of hantaviruses in Hubei Province. It has been suggested that genetic variation is ubiquitous in hantaviruses and that new genetic variants emerge constantly in the Hubei Province, which may influence the epidemic and transmission of the disease [21], [22]. The HFRS infection has been existing in Hubei Province for many years however, the epidemic characteristics of HFRS are still not entirely clear. Therefore, a systematic investigation of the spatial-temporal distribution pattern of HFRS is needed, in order to facilitate better prevention and control of HFRS in Hubei Province.

The current study aimed to characterize the spatiotemporal dynamic of the HFRS epidemic, to examine the changing patterns of HFRS in Hubei Province over a time span of the past 30 years (1980–2009) and to identify the probable reasons for changes in the epidemic trend of HFRS.

## Methods

### Study area

**Figure 1:**
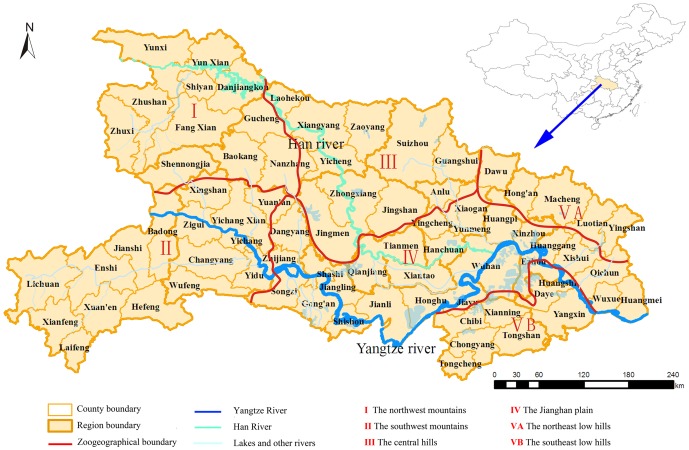
The location and division of study area, Hubei Province in Mainland China.

The study area is in Hubei (Figure 1), a province that is located in central China (29°05'–33°20'N, 108°21'–116°07'E). It encompasses an area of 189,500 square kilometers and has a population of more than 60 million. Hubei can be divided into the following five areas according to the terrain: the northwest mountains, the southwest mountains, the central hills, the Jianghan plain, and the northeast and southeast low hills. The east, west and north parts of Hubei are surrounded by mountains. The terrain lies ‘tilted’ from west to east, and forms an incomplete basin that is open to the south. There are 12 cities in Hubei that are directly managed by the provincial government, 1 autonomous prefecture, 1 forested region, 3 cities that are directly-managed, 21 cities that are managed at the county level and 39 counties. Administrative divisions have changed during the past 30 years, and some regions have been reorganized. In the current study, 76 counties are included in the analyses.

### Data collection

Cases of HFRS were initially diagnosed during their hospitalization according to the standard clinical criteria. Patient data and blood samples of diagnosed cases in local hospital were sent to corresponding-level Center for Disease Control and Prevention (CDC). The data and blood samples from all the local CDC in Hubei Province were collected by Hubei Provincial CDC. The infection of HFRS patients was confirmed by indirect IgM/IgG ELISAs using mixed antigens of HTNV and SEOV at the laboratory of Hubei Provincial CDC. The data were collected by case number, according to the sampling results and were processed by county and by month. Written informed consent was obtained from all patients and all analyzed data were anonymized. The Research Ethics Committee of Wuhan University approved this project.

A county-level polygon map (scale = 1∶4,000,000), including the 76 study counties, was obtained in order to conduct a GIS-based analysis on the spatial distribution of HFRS. Databases of geographical information and attributes were developed using the collected data. Attributes database consisted of disease data and demographic data. Disease data include HFRS cases per month and per year of every county in Hubei. All HFRS cases were geocoded according to administrative code and matched to the county-level layer of the polygon map using ArcGIS 10.0. Population data based on the 1990, 1995, and 2000 censuses was integrated in terms of the administrative code. The 1990 census records were used for population counts of the first decade studied (1980–1989). Population analyses for the periods 1990–1999 and 2000–2009 were based on census records from 1995 and 2000, respectively.

### General tendency and GIS mapping

Annual HFRS fluctuations in Hubei were examined by calculating and plotting the annual incidence of, and case fatalities due to HFRS between 1980 and 2009. Thematic maps were produced using ArcGIS 10.0 to illustrate the HFRS distribution in Hubei during each decade. All counties were grouped into four categories for the thematic maps as follows: non-endemic areas, low-level endemic areas with an annual average incidence between 0 and 5 per 100,000, moderate-level endemic areas with an annual average incidence between 5 and 30 per 100,000 and high-level endemic areas with an annual average incidence of over 30 per 100,000. Counties were color-coded on maps according to category.

### Space-time clustering analysis

Spatial scan statistical analyses were conducted using SaTScan to test for the presence and the location of HFRS clusters [23]. Scan statistics are used to detect and evaluate clusters in a temporal, spatial or space-time setting. This is done by gradually scanning a window across time and/or space, noting the cases observed and the expected observations at each location inside the window [24]. The window radius varies from 0 to a specified maximum value. The cluster assessment is done by comparing the number of cases within a window to the number of expected cases assuming that cases were randomly distributed in space. The likelihood ratio test is used to determine the significance of identified clusters and the p-value is obtained through Monte Carlo testing[25].

In the current study, retrospective space–time cluster analysis for higher incidence was used, in which the maximum spatial cluster size was set at 25% of the total population, and the maximum temporal cluster size was set at 50% of the total population. The null hypothesis of no clusters was rejected when the simulated p-value was less than or equal to 0.05.

### Seasonal analysis

The monthly incidence of HFRS was determined for all three decades and the five-year average monthly incidence of HFRS was calculated and plotted to examine monthly fluctuations of HFRS in Hubei. The incidence of HFRS in each county during the fall-winter was determined and the counties were divided into three categories according to incidence (<30%, 30–70%, >70%) in order to examine the seasonal distribution of HFRS. Counties were then mapped using different colors to illustrate season distribution. Fall-winter and spring-summer incidences for each county were shown in pie charts.

## Results

### Epidemic Curve

**Figure 2:**
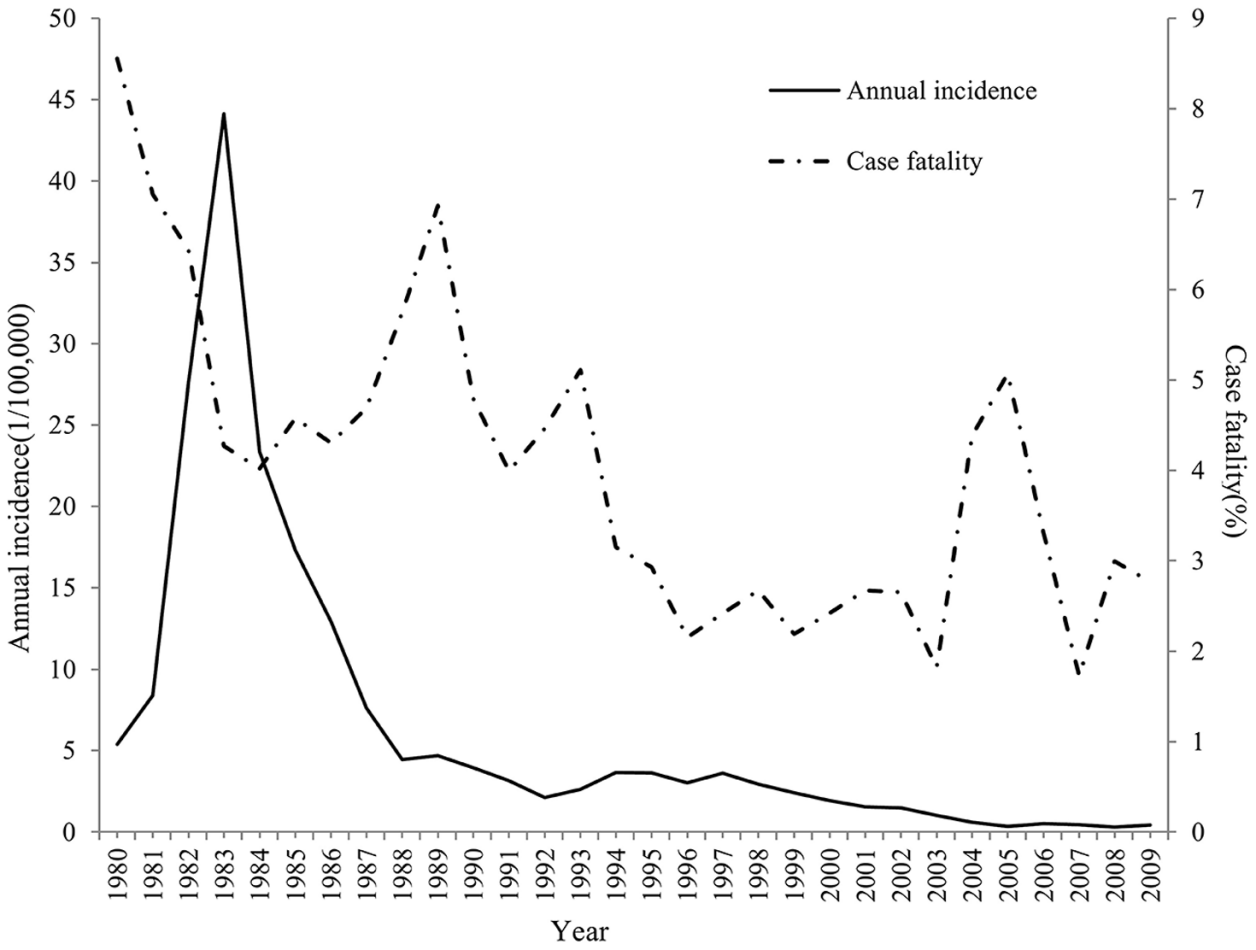
Annual incidence and case fatality of HFRS from 1980–2009 in Hubei Province, China.

A total of 104,467 HFRS cases were reported in Hubei between 1980 and 2009. The annual average incidence at the county level ranged from 0 to 655.04/100 000. An epidemic curve (Figure 2) shows the HFRS incidence and fatality trends for each year from 1980 through 2009. It is notable that, in 1983 the total number of cases was 23,943 and the incidence exceeded 40/100,000, which was the highest recorded incidence during the 30 years that were studied. The incidence of HFRS sharply decreased after that year. A fluctuating, but declining temporal trend in the annual incidence of HFRS was identified during 1990's. After 2000, incidence of HFRS gradually decreased and has remained below 1/100,000. The highest rate of fatalities due to HFRS was 8.31% and occurred in 1980. The lowest rate of fatalities was 0.4% occurred in 2007. The rate of fatalities due to HFRS decreased after 1980 and has remained at approximately 2.5% since the mid-nineties.

### Spatial distribution of HFRS in Hubei Province

**Figure 3:**
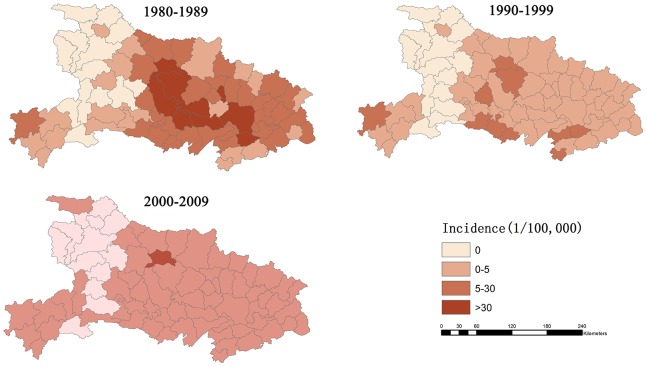
The spatial distribution of HFRS incidence in Hubei Province, China. The color gradient presents the annual incidence of HFRS in thematic maps. All counties were grouped into four categories: non-endemic area, low endemic area with annualized average incidence between 0 and 5 per 100,000, moderate endemic area with annualized average incidence between 5 and 30 per 100,000, and high endemic area with annualized average incidence over 30 per 100,000.

The epidemiology of HFRS for the period between 1980 and 2009 was analyzed in 76 counties of the Hubei Province. The spatial distribution of HFRS incidence during the three decades is shown on a thematic map (Figure 3) and the four different levels of endemic areas are displayed in gradient color. During the 1980's, 13 counties were considered to be non-endemic, 22 counties low-level endemic, 32 counties moderately endemic and 9 counties highly endemic. During the 1990's, 14 counties were considered to be non-endemic, 52 counties low-level endemic, 10 counties moderately endemic and no county was considered to be highly endemic. During the 2000's, 12 counties were considered to be non-endemic, 63 counties low-level endemic, 1 county was moderately endemic and no county was considered to be high-endemic.

### Cluster analysis of HFRS in Hubei

**Figure 4:**
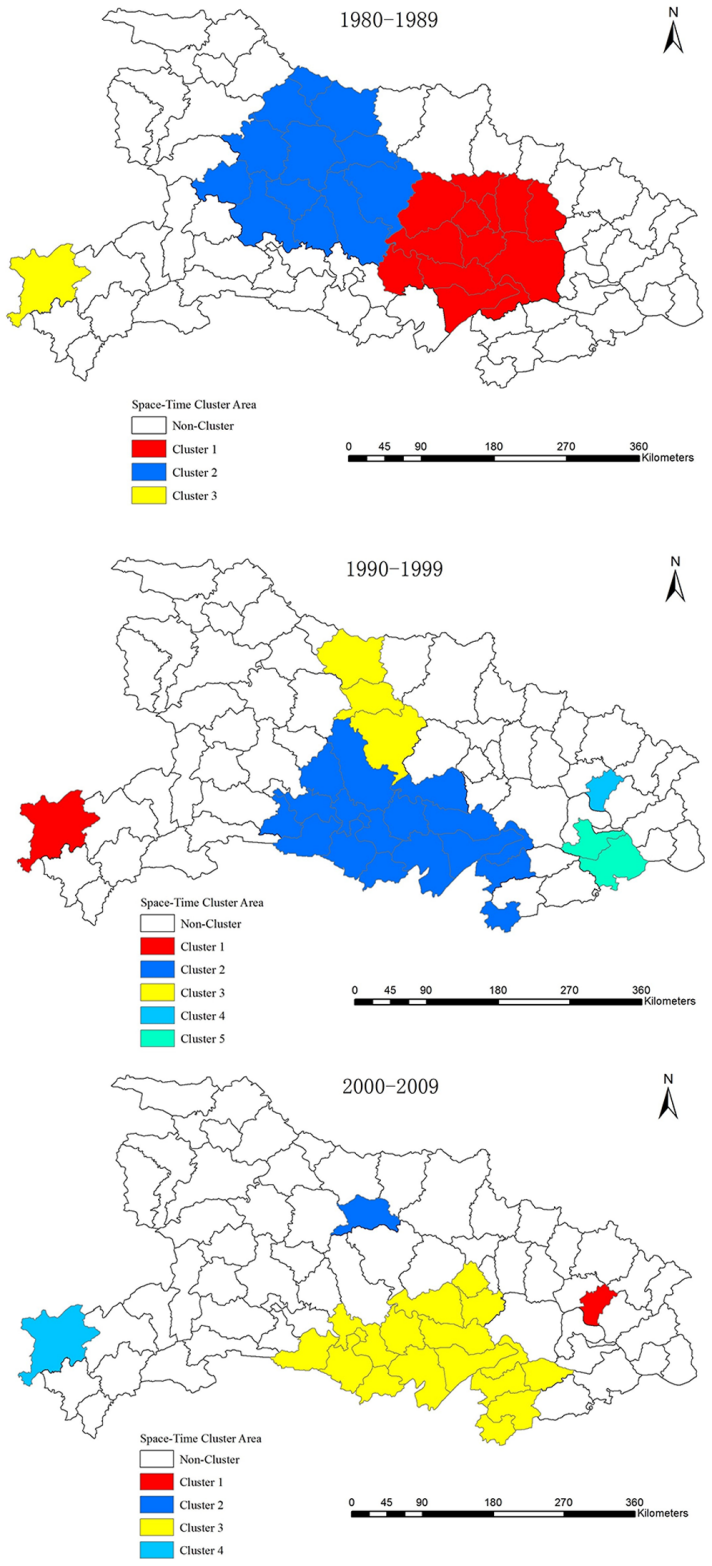
Space-time distribution of identified clusters of HFRS in Hubei Province, China. One most likely cluster and two secondary clusters were identified during the 1980's. The most likely cluster was comprised of 13 counties (Anlu, Jingshan, Xiaogan, Huangpi, Yingcheng, Yunmeng, Tianmen, Hanchua, Wuhan, Qianjiang, Xiantao, Jiayu and Honghu) for the period 1982–1985. One most likely cluster and four secondary clusters were identified during the 1990's. Lichuan was the most likely cluster from 1990 to 1994. One most likely cluster and three secondary clusters were identified in the 2000's. The most likely cluster area was found in Huanggang in 2000–2003.

Space-time cluster analysis of HFRS in Hubei showed that HFRS was not distributed randomly in space. The results of the spatial cluster analysis are summarized in Table 1 and Figure 4. One most likely cluster and two secondary clusters were identified during the 1980's. The most likely cluster was comprised of 13 counties (Anlu, Jingshan, Xiaogan, Huangpi, Yingcheng, Yunmeng, Tianmen, Hanchua, Wuhan, Qianjiang, Xiantao, Jiayu, and Honghu) located in the central part of Hubei in 1982–1985. One most likely cluster and four secondary clusters were identified during the 1990's. Lichuan, situated in the southwest area of Hubei, was the most likely cluster from 1990 to 1994. The spatial size of the 1990–1994 cluster was smaller than the 1982–1985 cluster. During the 1990's, cluster area locations had generally shifted to the south and east of Hubei compared with the 1980's. One most likely cluster and three secondary clusters were identified in the 2000's, however the cluster areas had decreased compared with the previous two decades. The most likely cluster area was found in Huanggang in 2000–2003. Notably, the Central hills and Jianghan plain regions, had consistently been detected to possess high-risk areas during these three decades, although the locations and scale of disease clusters had changed over time. Furthermore, Lichuan had been observed to be a high-risk area of HFRS throughout the whole period.

**Table 1:** Detection of HFRS Clusters in Hubei Province, China.

Time-frame	Cluster	Cluster date	LLR	Observed^a^	Expected^b^	RR	P-value
1980–1989	1	1982–1985	27145.13	33757	8150.17	6.18	<0.001
	2	1983–1985	3088.14	8840	3462.18	2.73	<0.001
	3	1981–1982	72.78	491	270.16	1.82	<0.001
1990–1999	1	1990–1994	2582.38	1637	140.55	12.63	<0.001
	2	1994–1998	1450.86	5302	2484.88	2.56	<0.001
	3	1993–1997	651.45	1696	626.06	2.87	<0.001
	4	1998–1999	331.50	327	51.39	6.46	<0.001
	5	1990–1991	172.89	367	116.21	3.20	<0.001
	6	1997	2.37	241	208.93	1.16	<0.001
2000–2009	1	2000–2003	829.66	422	23.94	19.21	<0.001
	2	2001–2005	577.73	329	23.11	15.20	<0.001
	3	2000–2002	482.54	1053	365.27	3.40	<0.001
	4	2000–2001	155.11	118	13.16	9.16	<0.001
	5	2000	0.23	72	66.42	1.09	= 1.000

LLR, Log likelihood ratio; RR, relative risk.

a Number of observed cases in a cluster.

b Number of expected cases in a cluster.

### Seasonality analysis of HFRS in Hubei

**Figure 5:**
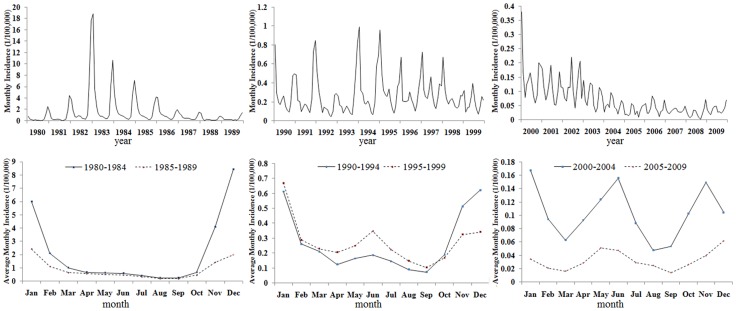
Temporal distribution patterns of HFRS incidence in Hubei Province. (A) Monthly epidemic curve of HFRS incidence for three decades. (B) Average monthly epidemic curve of HRFS cases every five years.

Seasonal epidemic curves were created to exhibit HFRS temporal fluctuations in Hubei between 1980 and 2009. Figure 5A shows the monthly incidence during the 1980's, 1990's and 2000's. Epidemic curves of the 5-year average monthly incidence are shown in Figure 5B. Seasonal epidemic patterns of HFRS have notably changed during the past 30 years. In the 1980's, hantavirus infection during the fall-winter season accounted for about 60–90% of the annual HFRS cases reported and a single epidemic peak occurred between November and February. In the 1990's, HFRS incidence during the fall-winter decreased and a small epidemic peak gradually began to appear between April and August. After 1995, an obvious bimodal epidemic pattern became apparent.

**Figure 6:**
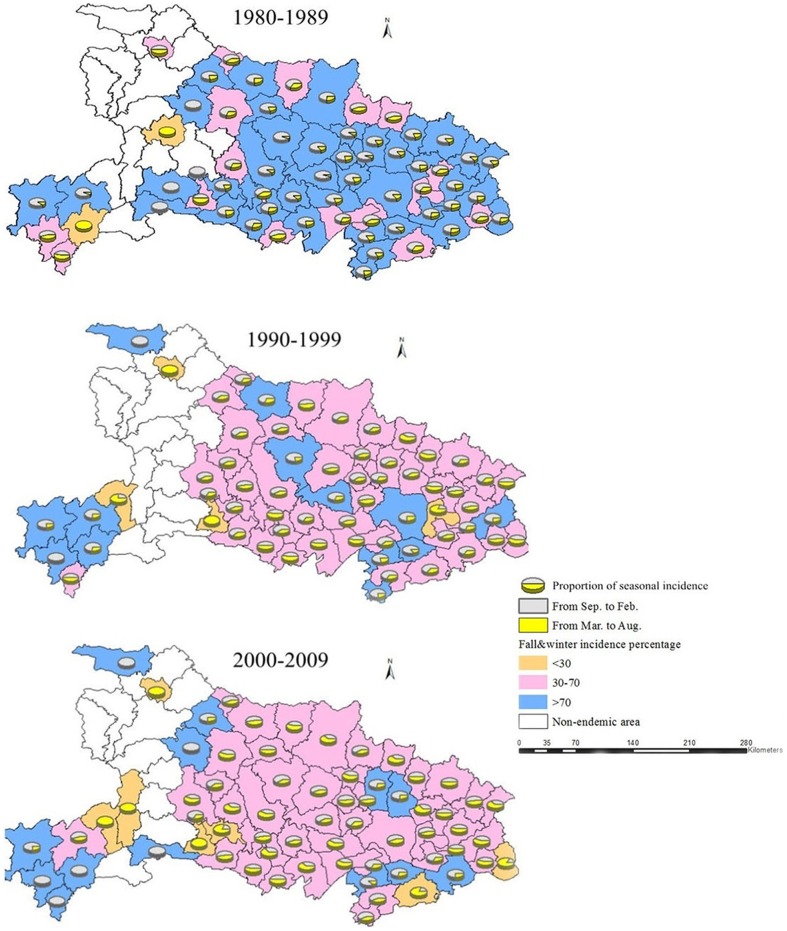
The seasonal incidence proportion of HFRS in each county for three decades. Different colored backgrounds present the fall-winter incidence proportional range of HFRS for each county. Pie graphs display the proportion of fall-winter and spring-summer incidence for each county. Counties in white have zero incidence.

The seasonal incidence, over three decades, for each county is shown in Figure 6. Districts shaded in blue show that the incidence of HFRS during the fall-winter accounts for more than 70% of annual cases. In the 1980's, the epidemic periods in most of the endemic areas appeared during the fall and winter. In the 1990's, the HFRS incidence during the spring and summer increased in almost every endemic area. By the 2000's, HFRS incidence in the spring and summer approached or exceeded the HFRS incidence in the fall and winter in about half of the counties studied. Thus, most of the HFRS endemic areas that were unimodal type epidemic areas (fall-winter peak) in 1980's had shifted to bimodal epidemic type areas.

## Discussion

HFRS has been a serious concern in Hubei for many decades. In the current study, we described the spatiotemporal distribution and seasonal patterns of HFRS in this region over a 30-year epidemic period (1980–2009). The HFRS incidence has had different epidemic characteristics over the past three decades. The incidence of and mortality due to HFRS have declined since the outbreaks that occurred during the 1980's. Additionally, HFRS incidence has become highly sporadic in recent years and a notable change in the seasonal epidemic pattern of HFRS has also been observed.

Hubei Province, known as the "land of a thousand lakes", mainly consists of alluvial plains and hills and has been a historical cultural center of China for 2500 years. The climate and ecological conditions in Hubei provide an ideal habitat for rodent populations. Our results demonstrate that HFRS has been endemic in all parts of Hubei with the main epidemic areas being the Jianghan plain and central hills regions. These areas are low in altitudes and have abundant water resources, with an annual average precipitation of 1100–1400 mm and 900–1100 mm, respectively. As shown in Figure 3 and 4, many areas of high HFRS incidence are distributed along the Han and Yangtze River. Furthermore, we found that many districts that contain or that are close to large lakes or rivers, such as Lichuan, Zhongxiang, Jingmen, Songzi, Gong'an, Shishou, Honghu, Qianjiang and Wuhan, have high incidences of HFRS during certain times. This phenomenon also mentioned in previous researches[26], [27], Bi Peng et al. found that most of the HFRS cases were distributed along river systems such as the Yangtze and the Huai in Anhui Province. Our results demonstrate that HFRS tend to occur or emerge in areas near large water systems, where the most suitable habitats for rodents are located. The geographical distribution of HFRS in Hubei suggests that environmental factors, such as terrain and water may play important roles in supporting rodent populations, which has increased high risk of HFRS in certain areas of Hubei.

HFRS was first reported in 1957 in Hubei and cases of HFRS increased rapidly, reaching a historically high level, in 1983. This period also had the highest provincial HFRS incidence in China. Our results clearly show that the incidence of, and case fatalities due to HFRS have decreased dramatically in Hubei during the past two decades, which is similar to the general trend in China [14]. Periodic outbreaks are one of the epidemiological characteristics of HFRS and the periodicity of HFRS is about 7–12 years [26]. In Hubei, two outbreaks occurred, one in the 1980's and the other in the 1990s. No further outbreaks of HFRS have occurred since the 1990's. The accumulated knowledge regarding this disease and improvements in diagnostics and therapeutic technology have greatly increased survival in cases of HFRS. There are several reasons for the decrease in HFRS incidence. First, after the 1980's outbreak, rodent control measures were implemented, such as rodent shelter and food source reduction in and around houses, preventing rodents from entering houses and removing rodents from houses, and using standard precautions for preventing hantavirus infection while rodent-contaminated areas are being cleaned. Second, urbanization, human migration from rural areas to cities,improvements in housing conditions and transformations in farm mechanization have all reduced exposure to wild rodents. Third, individuals in endemic areas or those who could be exposed are now vaccinated [7], [16], [28], [29].

In the current study, a notable shift of the seasonal epidemic patterns of HFRS during the past 30 years was observed. Seasonality is one of the epidemiologic characteristics of HFRS and the seasonal pattern is related to varying transmission dynamics of the two serotypes of hantaviruses. Early studies report that HTNV-related HFRS cases occur year-round but tend to peak in fall and winter while SEOV-related infections typically peak in spring, which are mainly transmitted by *A. agrarius* and *R. norvegicus* rodents, respectively [7], [9], [30], [31]. Given the peak periods of HFRS identified in our seasonal analysis, we infer that HFRS was primarily caused by HTNV in the 1980's and 1990's. This is consistent with previous findings indicating that the majority of patients with HFRS are infected with HTNV [32]. However, during the last decade, the HFRS has begun to occur more frequently in the spring and summer, accounting for about 50% of all cases. This suggests that the proportion of SEOV-related HFRS cases has greatly increased. This is consistent with the changing trend of epidemic patterns in Mainland China, where many endemic areas that were dominated by HTNV have become bimodal pattern endemic areas or SEOV dominant [9], [10], [33]. In our study, the sharp decrease over the last decade in the fall-winter incidence of HFRS could be responsible for the increase of proportion in HFRS incidence in spring. Thus, the spring peak highlighted is by the dramatic decline of HTNV-type infections during the fall-winter.

The mechanisms that drive the changing epidemiology of HFRS are complex and multifactorial [12], [27], [34], [35]. Factors influencing human-rodent interactions should be highly valued. Socioeconomic development has large influence on the transmission of hantaviruses to humans [14]. HTNV- and SEOV-related cases should be differentiated and analyzed separately since they are caused by two distinct rodent hosts with different habitats and lifestyles. HFRS has been found to be primarily caused by HTNV in Hubei, thus factors that influence HTNV-type HFRS should be taken into consideration. Since the launch of the reform and opening-up policy in 1980's, Hubei has experienced a fast economic transformation and has undergone large changes such as, agricultural development, urban construction, dam building, highway and railway construction, and irrigation engineering [36], [37], [38], [39]. These activities, as well as poor farming and construction conditions at that time, increased the likelihood of rodent-to-human contact in field and construction sites, and further facilitated the transmission of hantaviruses especially HTNV. Since the 1990's, raising standards of living, improved housing conditions and modified workplace conditions in the agriculture, forestry and construction industries has reduced exposure to *A. agrarius* rodents and further contributed to the decline of the annual incidence. The progression of urbanization and transformation of farm mechanization have also played positive roles in recent years.

Brown Norway rats are carriers of SEOV, and are the dominant species in human residential areas. Ecological surveillance showed that striped field mice mail live in fields, while Brown Norway rats live both in the field and houses[40], [41]. Brown Norway rats are spatially closer to human beings than striped field mice and more widely distributed in the world[42], [43]. HFRS cases that occur in urban areas are mainly caused by SEOV. It has been reported that, since the 1990's, new HFRS endemic areas in Mainland China are mainly SEOV-related and have a characteristic incidence peak that occurs in the spring [9], [17]. Contrary to its positive role in reducing the spread of HTNV-related disease, socioeconomic development can increase the risk for SEOV-related infection. For instance, urbanization and development of transportation systems can increase the frequency of migration for Norway rats, increasing the chances of contact with humans [15], [44]. Our previous research on the phylogenetic analysis of hantaviruses revealed three undefined lineages for SEOV and an SEOV spillover event in Hubei. The study concluded that Hubei is an significant epidemic focus for SEOV [21]. Considering that the majority of the trapped rodents in Hubei are Norway rats[21] and some areas of China are newly endemic for SEOV, the pathogenic role of SEOV should be taken into account in Hubei.

Our findings indicate that new epidemic characteristics of hantaviruses have emerged in Hubei. Wide dispersion of Brown Norway rats caused by rapid socioeconomic development and the emergence of new Seoul variants might subsequently lead to increase in the prevalence of SEOV infections in Hubei. Given this, vigilance in preventing SEOV-related HFRS should be reinforced and preventive measures in urban areas should be intensified. Health departments need to develop better prevention strategies and enhance the effectiveness of intervention when dealing with new epidemiological characteristics. Although seasonal distribution characteristics and the main type of animal host can preliminarily predict the epidemic type in affected areas, the prediction is not always completely accurate. Therefore, serological and molecular investigations should be done to confirm the serotype and genotype of hantaviruses in order to provide a more comprehensive and complete epidemiological analysis. In addition, further epidemiologic and ecologic studies are required to understand the exact variables that contribute to epidemic changes for each type of HFRS.
